# Glutathione *S*‐transferase omega class 1 (GSTO1)‐associated large extracellular vesicles are involved in tumor‐associated macrophage‐mediated cisplatin resistance in bladder cancer

**DOI:** 10.1002/1878-0261.13659

**Published:** 2024-05-15

**Authors:** Yi‐Cheng Pan, Pei‐Yi Chu, Ching‐Chan Lin, Ching‐Yun Hsieh, Wei‐Yu Hsu, Lie‐Fen Shyur, Juan‐Cheng Yang, Wei‐Chao Chang, Yang‐Chang Wu

**Affiliations:** ^1^ Ph.D. Program for Cancer Biology and Drug Discovery China Medical University and Academia Sinica Taichung Taiwan; ^2^ Chinese Medicine Research and Development Center China Medical University Hospital Taichung Taiwan; ^3^ Division of Hematology and Oncology, Department of Internal Medicine China Medical University, Hospital, China Medical University Taichung Taiwan; ^4^ Agricultural Biotechnology Research Center Academia Sinica Taipei Taiwan; ^5^ Graduate Institute of Integrated Medicine China Medical University Taichung Taiwan; ^6^ Ph.D. Program in Translational Medicine, College of Medicine Kaohsiung Medical University Taiwan; ^7^ Sex Hormone Research Center, Department of Obstetrics and Gynecology, Center for Molecular Medicine China Medical University Hospital Taichung Taiwan; ^8^ Center for Molecular Medicine China Medical University Hospital, China Medical University Taichung Taiwan; ^9^ Institute of Integrated Medicine, College of Chinese Medicine China Medical University Taichung Taiwan; ^10^ Department of Medical Laboratory Science and Biotechnology, College of Medical and Health Science Asia University Taichung Taiwan

**Keywords:** bladder cancer, cisplatin resistance, extracellular vesicle, GSTO1, tumor‐associated macrophage

## Abstract

Bladder cancer poses a significant challenge to chemotherapy due to its resistance to cisplatin, especially at advanced stages. Understanding the mechanisms behind cisplatin resistance is crucial for improving cancer therapy. The enzyme glutathione *S*‐transferase omega class 1 (GSTO1) is known to be involved in cisplatin resistance in colon cancer. This study focused on its role in cisplatin resistance in bladder cancer. Our analysis of protein expression in bladder cancer cells stimulated by secretions from tumor‐associated macrophages (TAMs) showed a significant increase in GSTO1. This prompted further investigation into the role of GSTO1 in bladder cancer. We found a strong correlation between GSTO1 expression and cisplatin resistance. Mechanistically, GSTO1 triggered the release of large extracellular vesicles (EVs) that promoted cisplatin efflux, thereby reducing cisplatin–DNA adduct formation and enhancing cisplatin resistance. Inhibition of EV release effectively counteracted the cisplatin resistance associated with GSTO1. In conclusion, GSTO1‐mediated EV release may contribute to cisplatin resistance caused by TAMs in bladder cancer. Strategies to target GSTO1 could potentially improve the efficacy of cisplatin in treating bladder cancer.

## Graphical abstract

In bladder cancer, the expression of glutathione *S*‐transferase omega 1 (GSTO1) can be induced by tumor‐associated macrophage‐secreted tumor necrosis factor‐alpha. The overexpression of GSTO1 causes cisplatin resistance through cisplatin efflux mediated by the generation of large extracellular vesicles. Targeting GSTO1 may represent a promising therapeutic strategy to enhance the efficacy of cisplatin treatments.

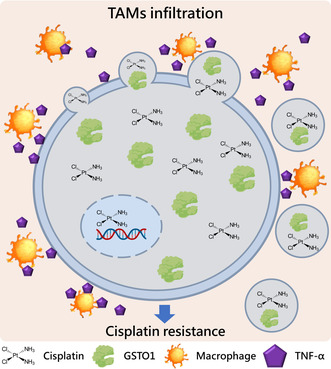

**Abbreviations**
ABC: ATP‐binding cassette
CD163: cluster of differentiation 163
CM: condition media
EVs: extracellular vesicles
FN1: fibronectin‐1
GFP: green fluorescent protein
GST: glutathione *S*‐transferase
GSTO1: glutathione *S*‐transferase omega class 1
HGUC: high grade urothelial carcinoma
HR: hazard ratio
*H*‐score: histochemistry score
ICP‐MS: inductively coupled plasma mass spectrometry
KO: knockout
LGUC: low‐grade urothelial carcinoma
MIBC: muscle‐invasive bladder cancer
MMP9: matrix metalloproteinase‐9
OE: overexpressed
OS: overall survival
PFS: progression‐free survival
PLAU: urokinase‐type plasminogen activator
Pt: platinum
TAMs: tumor‐associated macrophages
TME: tumor microenvironment
TNF‐α: tumor necrosis factor‐alpha

## 1 Introduction

Bladder cancer is the most commonly diagnosed cancer of the human urinary system and the most common malignancy, accounting for around 573 000 new cases and 213 000 deaths in 2020 [1]. Muscle‐invasive bladder cancer (MIBC), representing an advanced stage with an elevated risk of systemic spread, is associated with a high mortality rate among patients [2]. Cisplatin, a platinum (Pt)‐based drug, is the first‐line treatment for bladder cancer. It causes the generation of DNA lesions, followed by the induction of cell death. The formation of DNA adducts by cisplatin crosslinking with the purine bases of DNA is crucial. However, the efficacy of this therapy is limited due to drug resistance in advanced bladder cancer patients. Consequently, progression‐free survival (PFS) and overall survival (OS) are only 9 and 14 months, respectively [3, 4]. The development of cisplatin resistance is a complex process that may involve multiple factors, such as DNA repair, resistance to apoptosis, an inflammatory tumor microenvironment (TME), metabolism inside tumor cells, and drug‐resistant protein expression [5]. Tumor‐associated macrophages (TAMs) are the most abundant population in TME and are implicated in Pt‐based drug resistance in several cancers, including colorectal cancer [6] and ovarian cancer [7]. The TAM functions potentially involved in bladder cancer chemoresistance remain uncharacterized.

Glutathione *S*‐transferase omega class 1 (GSTO1) is a member of the cytosolic glutathione *S*‐transferase (GST) family. It is a multifunctional enzyme and is involved not only in xenobiotic detoxification and redox homeostasis [8], but also in the modulation of signaling pathways in several pathological conditions, including neurological disorders, inflammation, and cancers [9, 10]. The role of GSTO1 in cancer progression has attracted increasing attention over the past few years. Upregulation of GSTO1 expression is reported for diverse cancers, including esophageal squamous cell carcinoma [11], colorectal cancer [12], non‐small‐cell lung cancer [13], and bladder cancer [14], and is associated with metastatic features and advanced cancer stages. In addition, GSTO1 is suggested to be involved in chemoresistance in ovarian [15], colon [16], and cervical [17] cancers. The GSTO1 inhibitor sensitizes melanoma to cisplatin treatment [18]. However, the mechanism of GSTO1‐related drug resistance is still unclear. The increased presence of GSTO1 in bladder cancer has been linked to tumor progression [14]. However, the potential involvement of GSTO1 in drug resistance and its underlying mechanism within the realm of bladder cancer remain unexplored.

In some cancers, extracellular vesicles (EVs) are one of the causes of drug resistance. EVs are cell‐produced lipid‐capsuled membrane particles that possess biological functions, including the transportation of cell molecules from donor to recipient cells. The common transfer cargo molecules in EVs include proteins, nucleic acids, lipids, and metabolites [19]. Notably, GSTO1 is reported to be present in the EVs from endometrial cells [20], platelets [21], and TAMs [22], suggesting GSTO1 may play a role in the EVs. However, the precise role of GSTO1 in EVs remains unclear. In tumors, EVs may contribute to drug resistance in several ways, including sequestration of antitumor drugs, which prevents donor cell death, and transmission of drug efflux pumps and pro‐survival or anti‐apoptotic cargo to recipient cells, which increases the drug resistance of these cells [23]. Inhibition of EV generation by GW4869 prevents EV‐related drug resistance in pancreatic cancer [24] and ovarian cancer [25]. The inhibitors of EV uptake or knockdown dynamin 2 and clathrin also sensitize the cisplatin response in ovarian cancer cells [14, 26]. Although EV plays an important role in cancer progression, not all EV communication is pro‐tumorigenic. The targeting of deleterious EV subtypes is the main challenge to the control of EV‐related drug resistance.

The present study shows the possible role of GSTO1 in TAM‐mediated cisplatin resistance in bladder cancer. Our data indicate that TAMs are capable of inducing GSTO1 expression in bladder cancer by secreting tumor necrosis factor‐alpha (TNF‐α). The overexpression of GSTO1 leads to the acquisition of cisplatin resistance, as it facilitates cisplatin efflux by releasing large EVs from bladder cancer cells. These findings highlight the potential involvement of GSTO1‐associated EVs and the underlying effects of TAMs on the development of chemoresistance in bladder cancer.

## 2 Materials and methods

### 2.1 Chemicals

Phorbol 12‐myristate 13‐acetate (PMA, #10008014) was purchased from Cayman Chemical (Ann Arbor, MI, USA); 3‐(4,5‐dimethylthiazol‐2‐yl)‐2,5‐diphenyltetrazolium bromide (MTT; #M6494) was purchased from Invitrogen (Waltham, MA, USA); anti‐cisplatin DNA adduct antibody, clone ICR4 (#MABE416) was purchased from Merck (Darmstadt, Germany); cis‐diamineplatinum (II) dichloride (#479306), RPMI 1640 (#6504), and Y27632 (#SCM075) were purchased from Sigma‐Aldrich (St. Louis, MO, USA); SYTO 63 Red Fluorescent Nucleic Acid Stain (#S11345) and Zeocin (#R25005) were purchased from Thermo Fisher Scientific (Waltham, MA, USA). Granulocyte‐macrophage colony‐stimulating factor (GM‐CSF) and macrophage colony‐stimulating factor (M‐CSF) were purchased from Peprotech (Boston, MA, USA). Growth‐regulated oncogene alpha (GRO‐α), interleukin‐1α (IL‐1α), interleukin‐1β (IL‐1β), interleukin‐2 (IL‐2), interleukin‐6 (IL‐6), interleukin‐18 (IL‐18), macrophage inflammatory protein beta (MIP‐1β), stromal cell‐derived factor 1 alpha (SDS‐1α), TNF‐α, and vascular endothelial growth factor A (VEGF‐A) were purchased from CROYEZ (Taipei, Taiwan).

### 2.2 Cell line and cell culture

Human bladder cancer cell lines HT1376 (RRID: CVCL_1292), BFTC905 (RRID: CVCL_1083), and human leukemia monocytic cell line THP‐1 (RRID: CVCL_0006) were purchased from the American Type Culture Collection (ATCC, Manassas, VA, USA). All cells were used between passage numbers 15 and 45. HT1376 and BFTC905 were maintained in alpha Minimal Essential Medium (α‐MEM, #M0644; Sigma‐Aldrich) and Dulbecco's Modified Eagle Medium/Nutrient Mixture F‐12 Medium (DMEM/F‐12, #D8900; Sigma‐Aldrich), respectively. THP‐1 was grown in suspension in Roswell Park Memorial Institute 1640 Medium with GlutaMAX (RPMI/GlutaMAX, #61870036; Invitrogen) supplemented with 10% fetal bovine serum (FBS, #A5256701; Gibco, Waltham, MA, USA). All cell lines were grown in a humidified atmosphere of 5% CO_2_ and 95% air at 37 °C. The cisplatin‐resistant cells of HT1376 and BFTC905 cells were developed by stepwise increasing the cisplatin concentration from 0.05 to 10 μm. The multiplex PCR is performed using the AmpFLSTR Identifiler PLUS PCR Amplification Kit (#4427368; Applied Biosystems, Waltham, MA, USA), which contains 16 STR loci. The PCR products labeled with different fluorescence are analyzed with GeneMapper ID v3.1 on the capillary Genetic DNA analyzer 3730 (Applied Biosystems). The genotyping results of the sample are searched for in the human short tandem repeats (STR) profile database. The DSMZ, together with the ATCC, JCRB, RIKEN, ECACC, and ExPASy repositories, including data sets of more than 2455 cell lines, have generated comprehensive databases of STR cell line profiles. All of our cell lines had matching scores of 100% and were identical to the predicted cell line of origin, verifying the cell line's validity. We used sensitive technologies such as PCR to validate the absence of mycoplasma in our cell cultures on a regular basis, ensuring reliable results. In the cells mentioned above, no positive mycoplasma infection was found.

### 2.3 Induction of THP‐1 differentiation

The TME influences macrophage recruitment and polarization, causing M0 macrophages to differentiate into M2 [27]. To mimic the TEM, conditioned media was prepared by coculturing macrophages and tumor cells. THP‐1 cells were seeded onto the inserts of a transwell (24 mm diameter, 0.4 μm pore size, #3412; Corning, Glendale, AZ, USA) at a density of 1 × 10^6^ cells/insert. THP‐1 cells were induced to differentiate into macrophages by 100 ng·mL^−1^ PMA for 48 h and then maintained in a complete medium (RPMI 1640 supplemented with 10% FBS and 1% antibiotics) without PMA to recover for 24 h [28]. Meanwhile, HT1376 and BFTC905 cells were seeded in the 6‐well plate at a density of 5 × 10^5^ cells/well and allowed to attach overnight. Both macrophages and tumor cells were merged into one well and cocultured in a humidified atmosphere of 5% CO_2_ and 95% air at 37 °C for 24 h. After coculture, the culture media were collected and centrifuged at 3000 ***g*** for 10 min to remove the cell debris. After centrifugation, the supernatant was filtered through a 0.22 μm filter, resulting in the aliquoting and storage of the coculture conditional media (CM) at −30 °C for further use. The 10‐fold concentration of CM was prepared by centrifugation using 3K Macrosep Advance Centrifugal Devices (#MAP003C36; Pall Life Sciences, Washington, NY, USA), and the final 10% and 20% of concentrated CM were used in the experiments.

### 2.4 Establishment of GSTO1‐overexpressing (GSTO1‐OE) cells

The GSTO1/GFP overexpression cell line was established by the TransIT‐X2 dynamic delivery system (#MIR6000; Mirus, Madison, WI, USA). HT1376 cells were seeded into a 6‐well plate and grew to 70–90% cell density before transfection. The 2.5 μg pcDNA4‐GSTO1/GFP plasmid was mixed with 7.5 μL TransIT‐X2 transfection reagent in 250 μL OPTI‐MEM medium (Gibco) and incubated at room temperature for 15 min. The transfection mixture was added to the 6‐well plate, incubated at 37 °C for 24 h, and selected with 100 μg·mL^−1^ zeocin.

### 2.5 Generation of GSTO1‐knockout (GSTO1‐KO) cells

The GSTO1‐KO HT1376 cell line was constructed using CRISPR/Cas9 technology. Briefly, HT1376 cells were transfected with the Dharmacon Edit‐R Lentiviral Cas9 Nuclease Expression vector (GE Healthcare Dharmacon, Lafayette, CO, USA) and the gRNA targeting *GSTO1* (GAAGGCCAAG GGAATCAGGT; GE Healthcare Dharmacon) using the TransIT‐X2 system reagent. After transfection for 48 h, the cells were treated with 80 μg·mL^−1^ blasticidin (#15205, CAS: 3513‐03‐9; Sigma‐Aldrich) to select GSTO1‐KO cells. Western blotting was used to examine the expression of GSTO1 in GSTO1‐KO cells.

### 2.6 Bladder cancer xenograft animal assay

Animal experiments were performed in accordance with the guidelines and regulations at China Medical University, Taichung, Taiwan, and were approved by the Institutional Animal Care and Use Committee (IACUC) of China Medical University (animal protocol CMUIACUC‐2018‐213). Mice were maintained at a constant ambient temperature (23 ± 2 °C) under alternating 12‐h light/dark cycles and 50 ± 20% relative humidity in a pathogen‐free environment. They were free to access food and water. Twenty‐four male C.B17/lcr‐*Prkdc* ^*scid*^/CrlNarl mice, 5 weeks old and with severe combined immunodeficiency (SCID), were purchased from the National Laboratory Animal Center in Taipei, Taiwan. The experimental tumor cells (GSTO1‐OE or GSTO1‐KO) and the control HT1376 (1 × 10^6^ cells) were subcutaneously injected into the right and left flanks of SCID mice, respectively. Until tumor volume was approximately 100 mm^3^, mice were intravenously treated with cisplatin (3 mg·kg^−1^) 4 times per week. Tumor volume was calculated using the following equation: length × width × width/2. After four cycles of treatment, the mice were sacrificed using carbon dioxide (CO_2_), and these tumors were individually weighed. Inductively Coupled Plasma Mass Spectrometry (ICP‐MS) determined the intracellular Pt levels in the tumors.

### 2.7 *In silico* gene expression correlation analysis and survival analysis

The correlation between the expression levels of the target gene and overall survival (OS) of bladder cancer patients was analyzed by the Kaplan–Meier plotter server (http://kmplot.com/analysis/), which contained independent datasets from the cancer Biomedical Informatics Grid (caBIG), the Gene Expression Omnibus (GEO), and The Cancer Genome Atlas (TCGA) repositories. The high versus low expression levels of cluster of differentiation 163 (CD163) mRNA were split by the upper tertile value. The threshold of follow‐up for patients was set as 60 months. The hazard ratio (HR) was given with 95% confidence intervals, and the log rank *P* value was calculated and displayed on the webpage. The expression of the CD163 gene in various pathological stages was analyzed by the pathological stage plot in the Gene Expression Profiling Interactive Analysis (GEPIA) web server. The differential gene expression analysis was a one‐way ANOVA, using pathological stage as a variable for calculating differential expression. The expression violin plots were generated using log_2_ (TPM + 1) transformed expression data on the patient's pathological stage. Pair‐wise gene expression correlation analysis was performed at the GEPIA web server (http://gepia.cancer‐pku.cn/) using TCGA and the Genotype‐Tissue Expression (GTEx) expression data by a standard processing pipeline. The monotonic relationship between GSTO1, fibronectin‐1 (FN1), matrix metalloproteinase‐9 (MMP9), or urokinase‐type plasminogen activator (PLAU), and CD163 expression was calculated by the Spearman correlation coefficient.

### 2.8 Cisplatin–DNA adduct detection

The ability of cisplatin to form DNA adducts was evaluated using an anti‐cisplatin‐modified DNA antibody (#ab103261; Abcam, Cambridge, UK), which has a specific affinity for the cisplatin‐DNA adduct. A total of 1 × 10^6^ GSTO1‐OE and the control HT1376 cells were seeded and allowed to adhere for 24 h. After 10 μm cisplatin treatment for another 24 h, the cells were washed twice with PBS, trypsinized, and fixed in 70% ethanol at 4 °C overnight. Then the cells were incubated with 0.5 μg·mL^−1^ anti‐cisplatin‐modified DNA antibody in PBS containing 100 mg·mL^−1^ digitonin at 4 °C overnight. For the secondary antibody reaction, 5 μg·mL^−1^ goat anti‐rat IgG (H + L) antibody (#31430; Invitrogen) was used for 2 h at room temperature. The signals were analyzed using flow cytometry FACSCalibur (BD Biosciences, San Jose, CA, USA), and the data were analyzed using cell quest software (BD Biosciences); the fluorescent levels were expressed as the mean fluorescence intensity.

### 2.9 Cell viability assay

The effect of chemotherapeutic drugs and CM on cell viability was determined using the methylthiazol tetrazolium (MTT) method. Tumor cells were seeded into a 24‐well microplate at a density of 2 × 10^4^ cells/well and treated with five different doses of cisplatin (0, 1, 2, 5, 10 μm) for 24 h. After treatment, 200 μL of MTT solution (1 mg·mL^−1^ in PBS) was added to react for 4 h at 37 °C. The solution and 500 μL of dimethyl sulfoxide (DMSO) were removed to dissolve insoluble purple formazan dyes. Cell viability was calculated by the optical density (OD) at the wavelength of 570 nm, and the viability rate was defined as: cell viability (%) = (experiment OD_570_/control OD_570_) × 100%.

### 2.10 Western blot analysis

The expression levels of proteins were determined by SDS/PAGE separation and the following western blot assay. The protein concentration was determined using the Bradford assay. Large EVs were collected by centrifuging the supernatant after treating an equivalent number of wild‐type and GSTO1‐OE cells with cisplatin. The proteins from large EVs were then extracted using an equivalent volume of RIPA lysis and extraction buffer (Thermo Fisher Scientific). Forty micrograms of total protein were separated on 12% SDS‐polyacrylamide gel and transferred to a 0.45 μm polyvinylidene difluoride membrane (#HVWG04700; Millipore, Burlington, MA, USA) at 400 mA at ice for 3 h in 25 mm Tris–HCl, 197 mm glycine, and 13.3% (v/v) methanol. Membranes were blocked with 5% (w/v) skim milk in TBST for 1 h, then incubated with primary antibodies at 4 °C for 16 h. The membrane was rinsed three times for 15 min, each in TBST, and horseradish peroxidase‐conjugated secondary antibodies were added for reaction at room temperature for 1 h. After the same rinsing procedure, immunoreactive signals were revealed using an enhanced ECL substrate Western Lighting Plus‐ECL (#NEL103E001EA; PerkinElmer, Taipei, Taiwan) and recorded by developing photographic film under optimum exposure. The primary antibodies (diluted 1 : 1000) used in this study were anti‐GSTO1 antibody (#15124‐1‐AP; Proteintech, Rosemont, IL, USA), anti‐fibronectin antibody (#26836; Cell Signaling, Danvers, MA, USA), anti‐MMP9 antibody (#2270; Cell Signaling), anti‐PLAU antibody (#TA805243; OriGene, Rockville, MD, USA), anti‐CTR1 (#13086; Cell Signaling), anti‐CD9 antibody (#13174; Cell Signaling), anti‐TSG101 antibody (#72312; Cell Signaling), anti‐EGFR antibody (#4267; Cell Signaling), anti‐EpCAM antibody (#93790; Cell Signaling), anti‐EEA1 (#3288; Cell Signaling), anti‐β‐catenin (#9582; Cell Signaling), anti‐CAV1 (#3267; Cell Signaling), anti‐SQSTM1 (#23214; Cell Signaling) and anti‐β‐actin antibody (#4967; Cell Signaling). The secondary antibodies used in this study were anti‐mouse antibody (#7076; Cell Signaling) and anti‐rabbit antibody (#7074; Cell Signaling).

### 2.11 Transwell migration assay

HT1376 cells (0.5 × 10^5^ cells in 200 μL) were suspended in the upper half of a PET membrane transwell insert chamber (BD Biosciences) on a 24‐well plate. Media without FBS supplementation were added into the upper chamber, whereas media with 10% FBS supplementation were added into the lower chamber. After incubating at 37 °C for 24 h, the cells that had migrated through the insert were fixed with 3.7% formalin (Sigma‐Aldrich) and subsequently stained with 0.1% crystal violet (Sigma‐Aldrich). Crystal violet was extracted using 50% ethanol and 0.1% acetic acid for quantification and subjected to colorimetric measurement at 570 nm.

### 2.12 Colony formation assay

HT1376 cells were seeded into 6‐well plates (200 cells per well) and maintained at 37 °C in an incubator with 5% CO_2_. Cells were treated with EVs derived from conditional media of HT1376 cells treated with or without cisplatin (cisplatin untreated group served as the control) and maintained in culture for 1 week. Colonies were then fixed in formaldehyde (3.7%, v/v) and stained with crystal violet (0.5%, w/v). Colony formation was imaged and quantified spectrophotometrically at 570 nm after extraction in 50% ethanol and 0.1% acetic acid.

### 2.13 Pathological tissue array and bladder cancer tissue specimens

The expression of GSTO1 in human bladder normal tissues and tumor tissues was assessed using a pathological tissue array (#BL2081b) obtained from US Biomax in Derwood, MD, USA. This array comprises 142 cases of invasive urothelial carcinoma, 15 cases of squamous cell carcinoma, 12 cases of adenocarcinoma, 8 adjacent normal bladder tissues, and 8 normal tissues, with a single core per case. All tissue samples were sourced from certified hospitals that adhere to the following guarantees: (a) Informed Consent: All human tissue samples were and will continue to be collected with informed consent from the donors and their relatives, although the specific documents are not provided to US Biomax, Inc. (b) Medical Expertise: All tissue samples were and will be excised by licensed medical doctors. (c) Diagnosis and Identification: All tumor tissue samples were and will be diagnosed and identified by at least two different evaluators. The *H*‐score method was used to assess immunohistochemical results by converting the number of positive cells in each section and their staining intensity into corresponding values. The score value was obtained by the formula: [3 × (percentage of strongly staining cells) + 2 × (percentage of moderately staining cells) + 1 × (percentage of weakly staining cells)], giving a range of 0–300%.

The specimens were obtained from 13 patients diagnosed with bladder cancer who underwent cisplatin treatment at the China Medical University Hospital (Taichung, Taiwan). The specimens were collected from July 2008 to May 2012. These patients were classified based on their response to cisplatin treatment, with high responses indicating favorable outcomes and low responses indicating poor outcomes (cisplatin resistance). All patients provided written, informed consent. The study protocol (CMUH‐108‐REC2‐105) was approved by China Medical University Hospital, and the research was conducted in compliance with the Declaration of Helsinki.

### 2.14 Immunohistochemical assay

Immunohistochemical assay (IHC) was performed using an automatic BenchMark XT staining machine (Ventana Medical Systems, Tuscon, AZ, USA) and the iView 3,3‐diaminobenzidine (DAB) detection kit (Ventana Medical Systems). Paraffin sections (4 μm) containing human bladder cancer tissue specimens were deparaffinized, hydrated, and heated to 95–100 °C for 4 min to induce antigen retrieval. After inactivating endogenous peroxidase activity, rabbit anti‐human GSTO1 monoclonal antibody (1 : 200 dilution, #15124‐1‐AP; Proteintech) was used to perform IHC staining. Tissue sections were finally incubated with iView copper for 4 min to enhance signal intensity. Tissue specimens were counterstained with hematoxylin, dehydrated, mounted, and observed using an Eclipse E600 light microscope (Nikon, Minato‐ku, Tokyo). All staining results were evaluated by an experienced histologist.

### 2.15 Purification and size measuring of GSTO1‐containing large EVs

GSTO1‐OE HT1376 cells were treated with 5 μm cisplatin to induce EV release for 3 or 24 h. The GSTO1‐containing EVs were collected from the conditional media by a differential centrifugation method. Firstly, the solution was centrifuged at 300 ***g*** for 10 min to remove cell debris. Due to the large size of GSTO1‐containing EVs (10–20 μm in diameter), the supernatant was subjected to centrifugation at 3000 ***g*** for 30 min to collect EVs. After carefully removing the resultant supernatant, 100 μL of PBS was used to wash the precipitated EVs by gentle pipetting. Centrifugation was repeated at 3000 ***g*** for 30 min. The purified EVs were monitored by Cytation 5 (BioTek, Santa Clara, CA, USA), a fluorescence microscopy, and aliquoted for functional determination. The size of the GSTO1‐containing large EV was measured and quantified by the gen5 image ^+^ 3.10 version (BioTek).

### 2.16 Mass spectrometry (MS)‐based proteomic identification

CM‐induced BFTC905 proteomic alterations were identified by MS analysis. Total proteins of BFTC905 were extracted using RIPA lysis and extraction buffer (Thermo Fisher Scientific) and sonication. Protein concentration was determined using the Bio‐Rad protein assay kit by measurement of absorbance at 595 nm. The proteins (40 μg) of each sample were separated by 9.5% SDS/PAGE and divided into four gel fractions. After finely cutting (< 1 mm^3^), gel pieces were subjected to in‐gel digestion to produce tryptic peptides. The Orbitrap Exploris 480 mass spectrometer (Thermo Fisher Scientific) coupled with an Ultimate 3000 RSLC nanosystem (Thermo Fisher Scientific) was used for MS analysis. The tryptic peptides were separated by a capillary nanoViper EASY‐Spray C18 column system, and the MS instrument was operated in the positive ion mode with a spray voltage set to 1.85 kV in the data‐dependent acquisition mode. Top N multiple‐charged precursors were automatically isolated and fragmented according to their intensities within a cycle time of 3 s. A full MS scan was set at a resolution of 120 000 with an automatic gain control target of 300%, and an MS/MS scan was performed in the Orbitrap at a resolution of 30 000. Protein identification was performed using the proteome discoverer software v.2.4 (Thermo Fisher Scientific) with the SEQUEST HT search engine against the UniPort human protein sequence database with 1% FDR criteria using Percolator. Labeling‐free quantitation was performed using the functional node of the Precursors Ions Quantifier. The quantification of precursor abundance was based on peak intensities, and protein ratios were calculated based on the median of all possible pairwise peptide ratios. Gene ontology and Kyoto Encyclopedia of Genes and Genomes (KEGG) pathway enrichment were conducted using the National Cancer Institute Database for Annotation, Visualization, and Integrated Discovery. The proteomic datasets are available on the Japan ProteOme STandard Repository (jPOSTrepo) [https://repository.jpostdb.org/preview/96672705765d46747 b5910; Access key: 8426; Accession no. JPST002946 (PXD049719) and https://repository.jpostdb.org/preview/54 288402065d467684cec8; Access key: 8821; Accession no. JPST002945 (PXD049720)].

### 2.17 Inductively coupled plasma mass spectrometry (ICP‐MS)

Intracellular platinum (Pt) levels of cells/tissues were determined by high‐resolution inductively coupled plasma mass spectrometry (HR‐ICP‐MS; Element 2 HR‐ICP‐MS; Thermo Fisher Scientific). Cell/tissue samples were digested with 65% nitric acid (0.5 mL) and 30% hydrogen peroxide (0.5 mL) at 80 °C for 1 h. After cooling, a 25% ammonia solution (0.5 mL) was added to the digested solution to neutralize the excess nitric acid and subsequently diluted to the proper concentration with ultrapure water. The Pt calibration solutions were prepared in a 1% nitric acid solution with a concentration of 50–1000 ng·L^−1^. All diluted samples were analyzed by HR‐ICP‐MS with a resolution power of 10 000.

### 2.18 Statistical analysis

The data were displayed as the means ± SD or the mean ± SEM, and the significance of differences was examined by a two‐tailed Student's *t*‐test. Overall survival was determined by the Kaplan–Meier method, and the survival curves were compared using the log‐rank test. The statistical analysis of the data was performed using ibm spss statistics 22 (IBM, Armonk, NY, USA). A *P* value < 0.05 was considered statistically significant.

## 3 Results

### 3.1 TAMs are associated with poor chemotherapeutic response in patients with bladder cancer

**Fig. 1:**
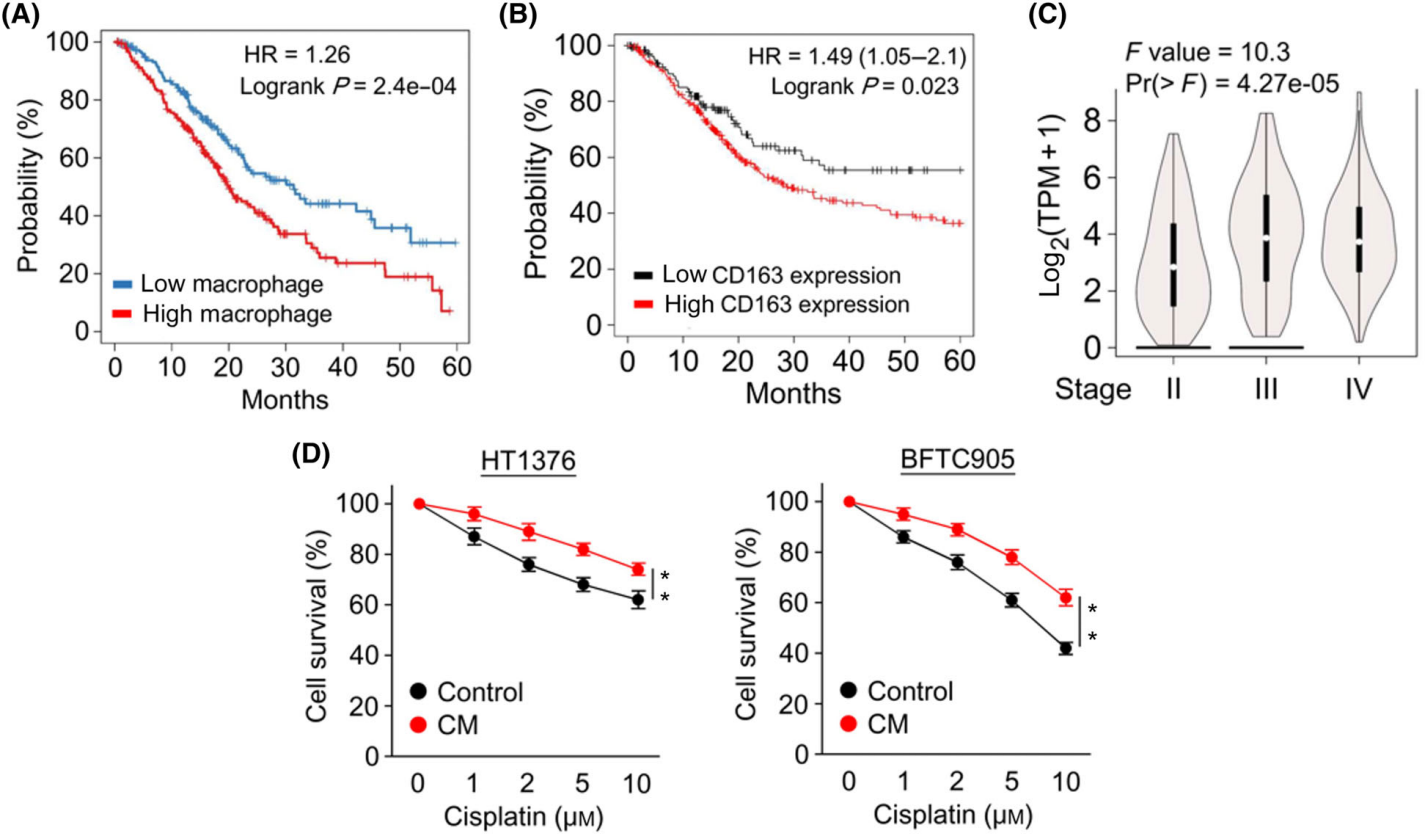
Macrophages increase the resistance of bladder cancer to cisplatin. (A) The correlation between macrophage infiltration and OS in patients with bladder cancer was analyzed by the TIMER2.0 website. (B) The correlation between CD163 expression and OS in patients with bladder cancer was analyzed by the Kaplan–Meier‐plotter cancer database. The high versus low expression levels of CD163 mRNA were split by the upper tertile value. The threshold of follow‐up for patients was set at 60 months. (C) The expression of the CD163 gene in various pathological stages of bladder cancer was analyzed by the pathological stage plot on the GEPIA web server. The expression violin plots were generated using log_2_ (TPM + 1) transformed expression data on the patient's pathological stage. (D) Cell viabilities of HT1376 and BFTC905 with or without 20% CM treatment in indicated doses of cisplatin for 24 h were determined by the MTT assay (*n* = 3). The data were displayed as the means ± standard deviation (SD). *P* values were calculated using a two‐tailed unpaired Student's *t*‐test (D). **P < 0.01. CM, condition media; OS, overall survival; TPM, transcripts per million.

Emerging evidence shows that TAMs potentially provide tumor‐supporting functions in the TME, thereby hampering the therapeutic response [29]. We used the TIMER2.0 website to analyze the clinical relevance of macrophage infiltration and the survival outcomes of bladder cancer patients [30]. The results showed a significant association between higher macrophage infiltration and poorer patient outcome, with a hazard ratio (HR) of 1.26 in the Cox proportional hazards model and a log‐rank *P*‐value of 2.4 × 10^−4^ for the Kaplan–Meier curve (Fig. 1A). CD163, a representative marker of macrophages, is usually used to determine TAM levels in the TME [31]. The results of the analysis of the Kaplan–Meier plotter cancer database [32] revealed that the OS of patients with bladder cancer was significantly inversely correlated with CD163 levels (Fig. 1B). The two results are consistent in suggesting that macrophage infiltration may aggravate bladder cancer. In addition, the CD163 expression levels were significantly correlated with the pathological stage of bladder cancer in the GEPIA website analysis [33] (Fig. 1C). To further assess whether TAMs impact the efficacy of cisplatin in treating bladder cancer, CM was used to mimic the interaction between macrophages and tumor cells in the TME [34] in the MIBC cell lines HT1376 and BFTC905. CM treatment increased the resistance of both cell lines to cisplatin in the MTT assay (Fig. 1D), suggesting that TAMs may confer cisplatin resistance on bladder cancer.

### 3.2 TAM secretion of TNF‐α enhances GSTO1 expression in bladder cancer

**Fig. 2:**
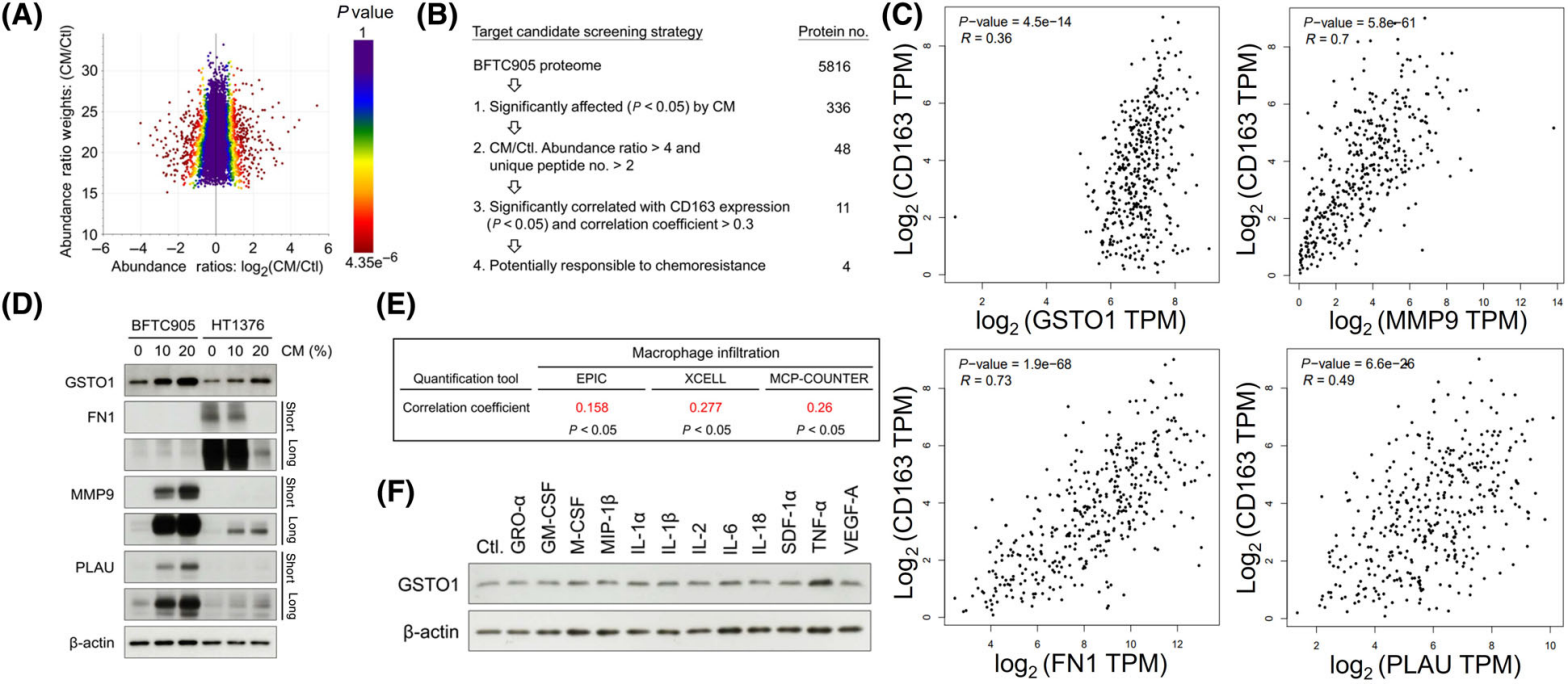
Macrophage secretory TNF‐α enhances GSTO1 expression in bladder cancer. (A) The proteomic alteration between CM‐treated and the control BFTC905 was analyzed using an MS‐based approach and shown by the scatter plot, which revealed the relationship between ratio weights (weighting by mass intensity) and abundance ratios of each protein. (B) The flowchart of the screening strategy for candidates responsible for chemoresistance from the proteomic analysis. (C) The correlation between GSTO1, FN1, MMP9, or PLAU and CD163 gene expression was analyzed on the GEPIA web server using the TCGA RNA‐Seq GTEx databases. (D) The expression of GSTO1, FN1, MMP9, and PLAU in HT1376 and BFTC905 with or without indicated doses of CM treatment was determined by western blot assay (*n* = 3). Short and long exposures were used to observe differential expression of proteins in both cells. (E) The correlation between GSTO1 expression and macrophage infiltration was estimated by EPIC, xCell, and MCP‐counter algorithms on the TIMER2.0 website. (F) The expression of GSTO1 in HT1376 treated with diverse cytokines was determined by western blot assay (*n* = 3). β‐Actin, loading control. The correlation coefficients were calculated by Spearman's correlation analysis (C, E). *P* values were calculated using Fisher's exact test (C, E). CM, condition media; Ctl, control; MS, mass.

To identify the critical molecules responsible for TAM‐mediated chemoresistance, we performed a comparative proteomic analysis to determine the protein alteration in BFTC905 under CM treatment. A total of 5816 proteins were identified in this analysis (Table S1). The proteomic change is shown in a scatter plot, which reveals the relationship between ratio weights (weighting by mass intensity) and abundance ratios of each protein; the color of the dot for protein represents the *P*‐value of the abundance ratio adjusted from the background *t*‐test (Fig. 2A). The potential candidates were further narrowed down by the following screening strategy (Fig. 2B): (a) a significantly different expression between the CM group and the control with an abundance ratio adjusted *P*‐value < 0.05 (336 eligible proteins); (b) abundance ratio CM/control > 4 and the identified protein with a unique peptide number > 2 (48 eligible proteins); and (c) a significant correlation with CD163 expression and a correlation coefficient > 0.3 indicating a moderate correlation [35]. Eleven proteins remained eligible for this criterion (Table 1; Table S2). The correlation plotters are selectively shown in Fig. 2C. Among these proteins (Table 1), FN1, MMP9, PLAU, and GSTO1 have been implicated in the promotion of chemoresistance in tumors [36, 37, 38, 39]. The expression of these proteins affected by CM treatment was validated by a Western blot assay. The expression of GSTO1 induced by CM in the two cell lines was more similar than the expression of the other three proteins, and the induction was in a dose‐dependent manner (Fig. 2D). GSTO1 expression showed a significantly positive association with the levels of infiltrating macrophages, with correlation coefficients of 0.158, 0.277, and 0.26 in the EPIC, xCell, and MCP‐counter algorithm analyses, respectively, on the TIMER2.0 website [30] (Fig. 2E). In addition, previous studies have reported the involvement of GSTO1 in cisplatin resistance in colon cancer [16]. However, the role of GSTO1 in drug resistance, specifically in bladder cancer, has never been investigated. Therefore, in this study, we focused on exploring the roles of GSTO1 in chemoresistance in bladder cancer. Accumulating evidence suggests that macrophage‐secreted cytokines play an important role in TAM‐mediated chemoresistance [34, 40, 41]. To determine the potential cytokines responsible for GSTO1 induction in bladder cancer, HT1376 was treated with individual cytokines, including GRO‐α, GM‐CSF, M‐CSF, MIP‐1β, IL‐1α, IL‐1β, IL‐2, IL‐6, IL‐18, SDF‐1α, TNF‐α, and VEGF‐A. Western blotting indicated the dominant effect of TNF‐α on GSTO1 induction in HT1376 (Fig. 2F).

**Table 1:** CM‐induced proteins that are potentially involved in chemoresistance in bladder cancer.

Protein name	Gene name	Spearman correlation		No. unique	MW (kDa)	Calc. pI	Abundance ratio	
		*P*‐value	*R*				CM/Ctl.	Adj. *P*‐value
Fibronectin	FN1	1.9E‐68	0.73	57	272.2	5.50	6.12	2.95E‐10
Matrix metalloproteinase‐9	MMP9	5.8E‐61	0.70	28	78.4	6.06	42.12	4.35E‐16
Filamin‐C	FLNC	5.5E‐37	0.58	87	290.8	5.97	8.14	1.81E‐13
Urokinase‐type plasminogen activator	PLAU	6.6E‐26	0.49	18	48.5	8.41	5.60	3.52E‐09
Leukocyte elastase inhibitor	SERPINB1	5.5E‐21	0.44	22	42.7	6.28	5.52	2.13E‐08
Protein Smaug homolog 1	SAMD4A	< 1E‐307	0.43	3	79.4	8.32	100.00	4.35E‐16
Glutathione *S*‐transferase omega‐1	GSTO1	4.5E‐14	0.36	20	27.5	6.60	6.01	4.35E‐16
Dehydrogenase/reductase SDR family member 9	DHRS9	2.0E‐13	0.35	15	35.2	8.60	12.20	4.35E‐16
TNF receptor‐associated factor 1	TRAF1	1.1E‐12	0.34	4	46.1	6.11	100.00	4.35E‐16
Putative uncharacterized protein MYH16	MYH16	9.1E‐11	0.32	36	128.2	5.49	14.46	4.35E‐16
Interferon‐stimulated gene 20 kDa protein	ISG20	4.8E‐10	0.30	8	20.4	8.92	7.64	3.16E‐07

### 3.3 GSTO1 contributes to cisplatin resistance in bladder cancer

**Fig. 3:**
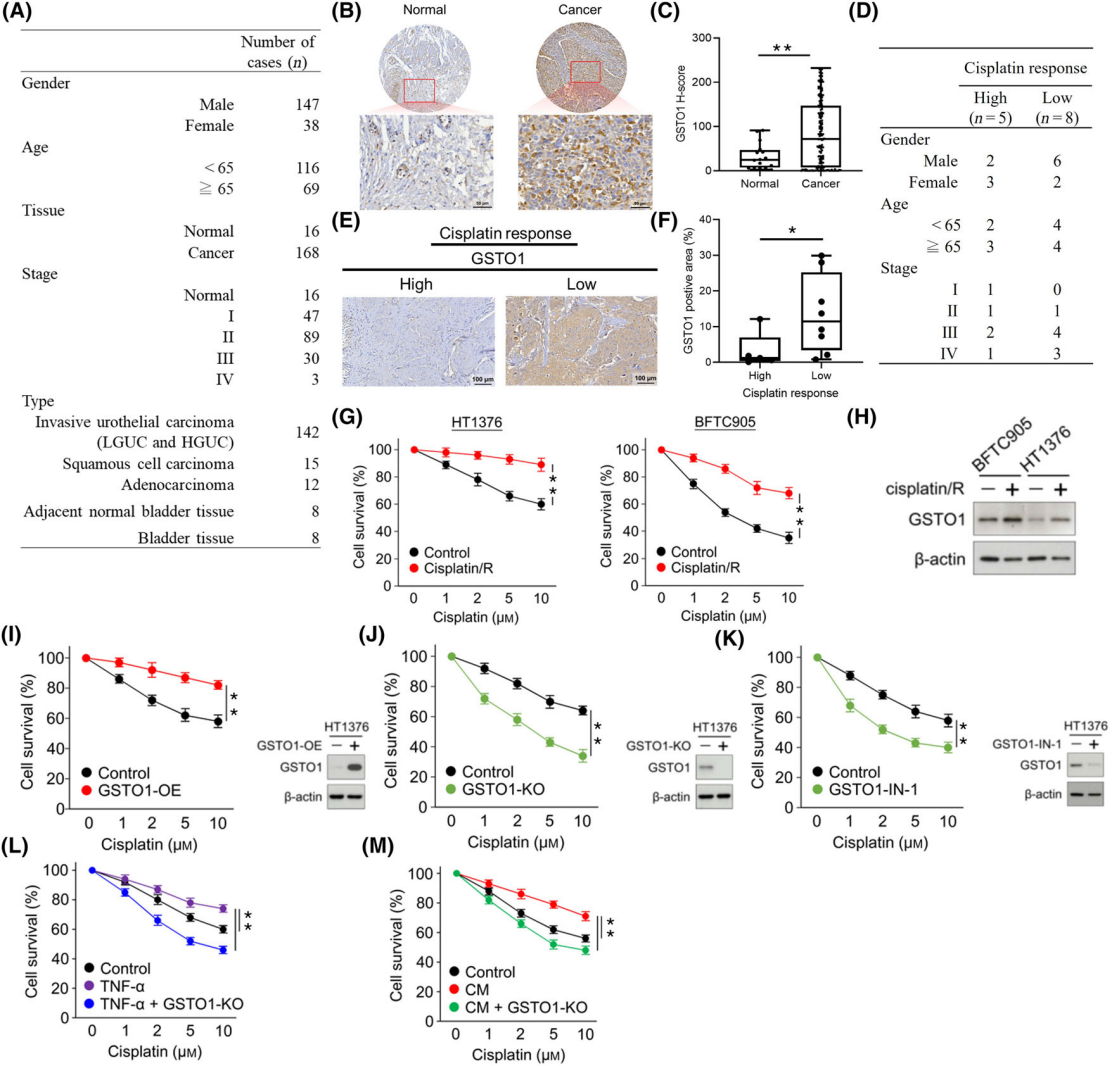
GSTO1 contributes to cisplatin resistance in bladder cancer. (A) The sample description of the pathological tissue array (Cat. No. BL2081b; US Biomax). (B) The expressions of GSTO1 in bladder normal (*n* = 16) and cancer tissues (*n* = 168) were analyzed using the pathological tissue array. Scale bar = 50 μm. (C) The *H*‐score method was used to quantitatively assess the IHC results (normal = 16; cancer = 168). (D) The sample description of bladder cancer patients after cisplatin treatment. (E) The expressions of GSTO1 in high (*n* = 5) and low (*n* = 8) cisplatin response patients using IHC. Scale bar = 100 μm. (F) The GSTO1 positive area was used to quantitatively assess the IHC results (low response = 8; high response = 5). (G) Cell viabilities of cisplatin‐resistant HT1376 and BFTC905 and their controls at indicated doses of cisplatin for 24 h were determined by the MTT assay (*n* = 3). (H) The expression of GSTO1 in cisplatin‐resistant HT1376 and BFTC905 and their controls was determined by western blot assay (*n* = 3). β‐Actin, loading control. (I) Cell viability of GSTO1‐OE and the control HT1376 at the indicated doses of cisplatin for 24 h was determined by the MTT assay. GSTO1‐OE in HT1376 was validated by western blot assay (right panel) (*n* = 3). (J) Cell viability of GSTO1‐KO and the control HT1376 at the indicated doses of cisplatin for 24 h was determined by the MTT assay. GSTO1‐KO in HT1376 was validated by Western blot assay (right panel) (*n* = 3). (K) Cell viabilities of HT1376 with or without GSTO1 inhibitor GSTO1‐IN‐1 (1 μm) treatment in indicated doses of cisplatin for 24 h were determined by the MTT assay (*n* = 3). The effect of GSTO1‐IN‐1 on GSTO1 expression of HT1376 was determined by Western blot assay (right panel) (*n* = 3). (L) Cell viabilities of GSTO1‐KO and the control HT1376 in the presence or absence of CM (20%) in indicated doses of cisplatin for 24 h were determined by the MTT assay (*n* = 3). (M) Cell viabilities of GSTO1‐KO and the control HT1376 in the presence or absence of TNF‐α (20 ng·mL^−1^) in indicated doses of cisplatin for 24 h were determined by the MTT assay (*n* = 3). Data were displayed as the means ± SEM (C, F) or the mean ± SD (G, I–M). *P* values were calculated using a two‐tailed, unpaired Student's *t*‐test (C, F, G, I–M). *P < 0.05; **P < 0.01. cisplatin/R, cisplatin resistance; HGUC, high‐grade urothelial carcinoma; IHC, immunohistochemistry; KO, knockout; LGUC, low‐grade urothelial carcinoma; OE, overexpression.

The bladder cancer tissue array analysis (*n* = 185) resulted in a higher GSTO1 histochemistry score (*H*‐score) for bladder tumors (*n* = 169) than for normal bladder tissue (*n* = 16) (Fig. 3A–C). To investigate the role of GSTO1 in cisplatin resistance in bladder cancer, GSTO1 expression was examined in 13 bladder cancer patients who underwent cisplatin treatment. Of these patients, eight experienced recurrences (indicating a low cisplatin response), while five did not (indicating a high cisplatin response). The data revealed that patients with recurrent bladder cancer after cisplatin treatment exhibited higher levels of GSTO1 expression (Fig. 3D–F). In addition, GSTO1 expression in cisplatin‐resistant and parental (cisplatin‐responsive) HT1376 and BFTC905 cells was determined. GSTO1 expression was higher in resistant cells than in parental cells (Fig. 3G,H). These results suggest that GSTO1 may contribute to the severity and cisplatin resistance of bladder cancer. To elucidate the impact of GSTO1 on cisplatin resistance, GSTO1‐overexpressed (GSTO1‐OE) and GSTO1‐knockout (GSTO1‐KO) cells were generated with HT1376 cells, and a GSTO1 inhibitor was employed. Under cisplatin treatment, GSTO1 overexpression increased the ability of bladder cancer to resist cisplatin cytotoxicity (Fig. 3I). On the other hand, GSTO1 knockout (Fig. 3J) and treatment with a GSTO1 inhibitor (Fig. 3K) sensitized bladder cancer cells to cisplatin cytotoxicity. Moreover, GSTO1 knockout rescued CM‐ and TNF‐α‐induced cisplatin resistance in HT1376 (Fig. 3L,M). Collectively, these results suggest functional roles for GSTO1 in both intrinsic and acquired cisplatin resistance in bladder cancer.

### 3.4 GSTO1 enhances cisplatin resistance by activating EV release

**Fig. 4:**
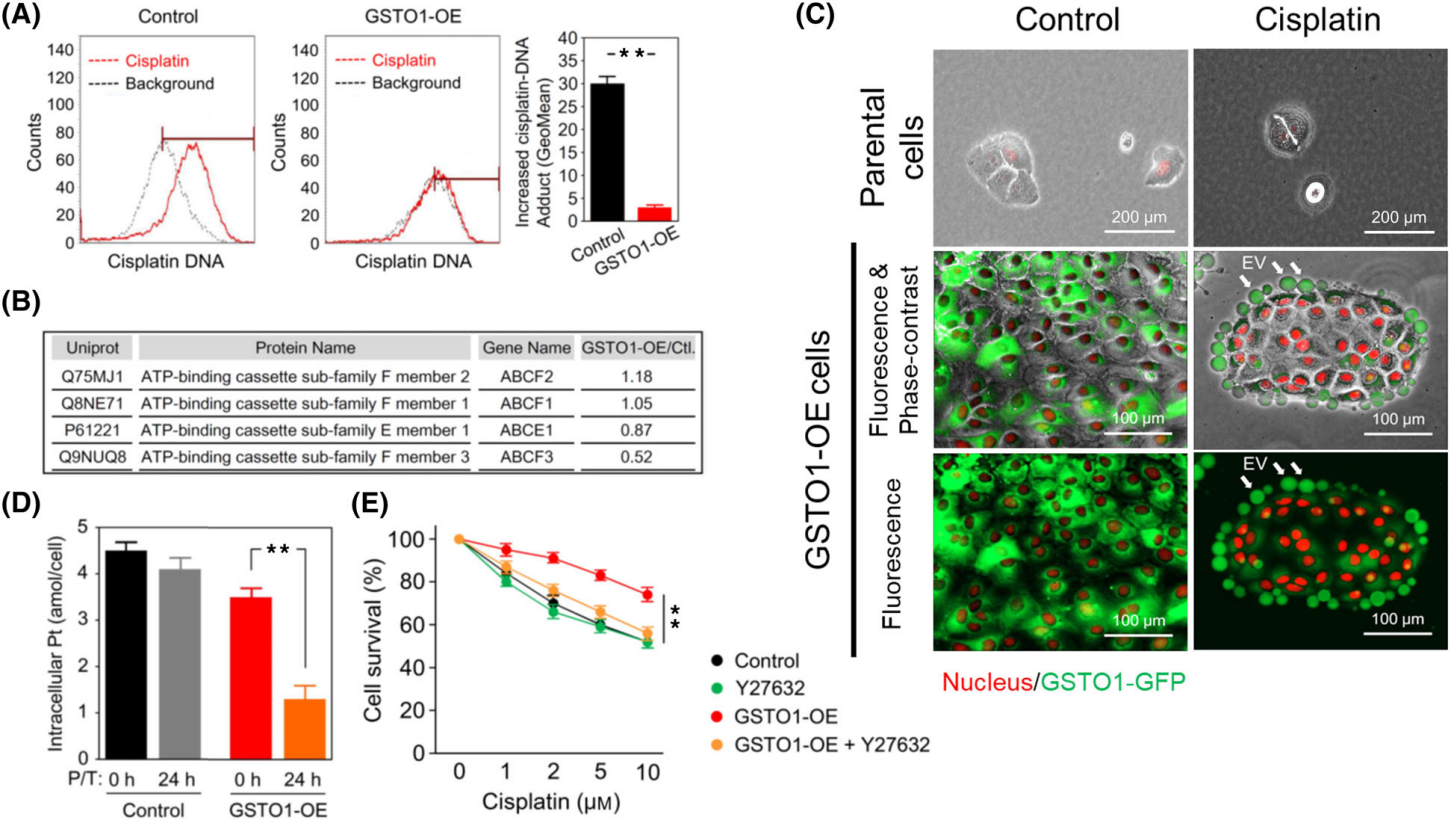
GSTO1 promotes cisplatin resistance by activating the release of EVs. (A) Intracellular DNA‐cisplatin adducts of GSTO1‐OE and the control HT1376 were analyzed using the anti‐cisplatin‐modified DNA antibody (Cat. No. ab103261; Abcam) by flow cytometry. The increasing levels of DNA‐cisplatin adducts compared to the background in the individual group were represented using differentially fluorescent signals as the mean fluorescence intensity (right panel) (*n* = 3). The H bar represents the region of cisplatin positive signals. (B) Alterations of the ABC transporter levels between GSTO1‐OE and the control HT1376 in comparative proteomic analysis. (C) After cisplatin (5 μm) treatment for 3 h, GSTO1‐OE and the control HT1376 were examined by phase‐contrast and fluorescence microscopy. The formation of EVs (indicated by the arrow) with a diameter larger than 10 μm dramatically appeared in the GSTO1‐OE cells (*n* = 3). (D) GSTO1‐OE and the control HT1376 were treated with cisplatin (5 μm) for 6 h; these cells were defined as 0 h P/T. After washing and culturing in cisplatin‐free media for another 24 h, the tumor cells were defined as 24 h P/T. The intracellular Pt levels were determined by ICP‐MS (*n* = 3). (E) Cell viabilities of GSTO1‐OE and the control HT1376 with or without EV inhibitor Y27632 (20 μm) treatment were determined by the MTT assay (*n* = 3). Data were displayed as the mean ± SD. *P* values were calculated using a two‐tailed unpaired Student's *t*‐test (A, D, E). **, *P* < 0.01. post‐treatment (P/T); platinum (Pt); Inductively Coupled Plasma Mass Spectrometry (ICP‐MS).

To decipher the underlying mechanisms of GSTO1's promotion of cisplatin resistance, intracellular DNA–cisplatin adducts and transporter proteins were determined. GSTO1 overexpression significantly attenuated the formation of DNA–cisplatin adducts compared to control cells (Fig. 4A). This suggests that the control of cisplatin levels in cells could be the key to GSTO1‐associated cisplatin resistance in cancer cells. The cisplatin level in cells is controlled by both its efflux and uptake mechanisms. ATP‐binding cassette (ABC) transporters are known to cause the efflux of chemotherapeutic drugs in cancer [42], while copper transporter 1 is the principal gateway for the entrance of cisplatin into cancer cells [43]. However, there were no significant differences in the levels of expression of ABC transporters, including ABCF2, ABCF1, ABCE1, and ABCF3 (Fig. 4B), between GSTO1‐OE and parental cells. To facilitate observations, GSTO1 was expressed with green fluorescent protein (GFP Tag) in GSTO1‐OE HT1376 cells. Surprisingly, under cisplatin treatment, the production of EVs containing GFP‐labeled GSTO1 was clearly observed in GSTO1‐OE cells but not in parental cells (Fig. 4C). Cisplatin efflux was evaluated by measuring the intracellular cisplatin content in tumor cells after removing the cisplatin treatment. GSTO1‐OE significantly reduced the intracellular levels of Pt content compared to parental cells (Fig. 4D). EV formation could be crucial for GSTO1‐associated cisplatin resistance. To verify the relationship between EV release and cisplatin resistance, we added Y27632 [44], a pharmaceutical inhibitor of EV release, to GSTO1‐OE HT1376 cells. As expected, the addition of Y27632 reversed the cisplatin resistance induced by GSTO1 overexpression (Fig. 4E). Taken together, these results suggest that GSTO1 enhances cisplatin resistance in bladder cancer by activating EV release to efflux intracellular cisplatin.

### 3.5 Characterization of GSTO1‐associated EVs

**Fig. 5:**
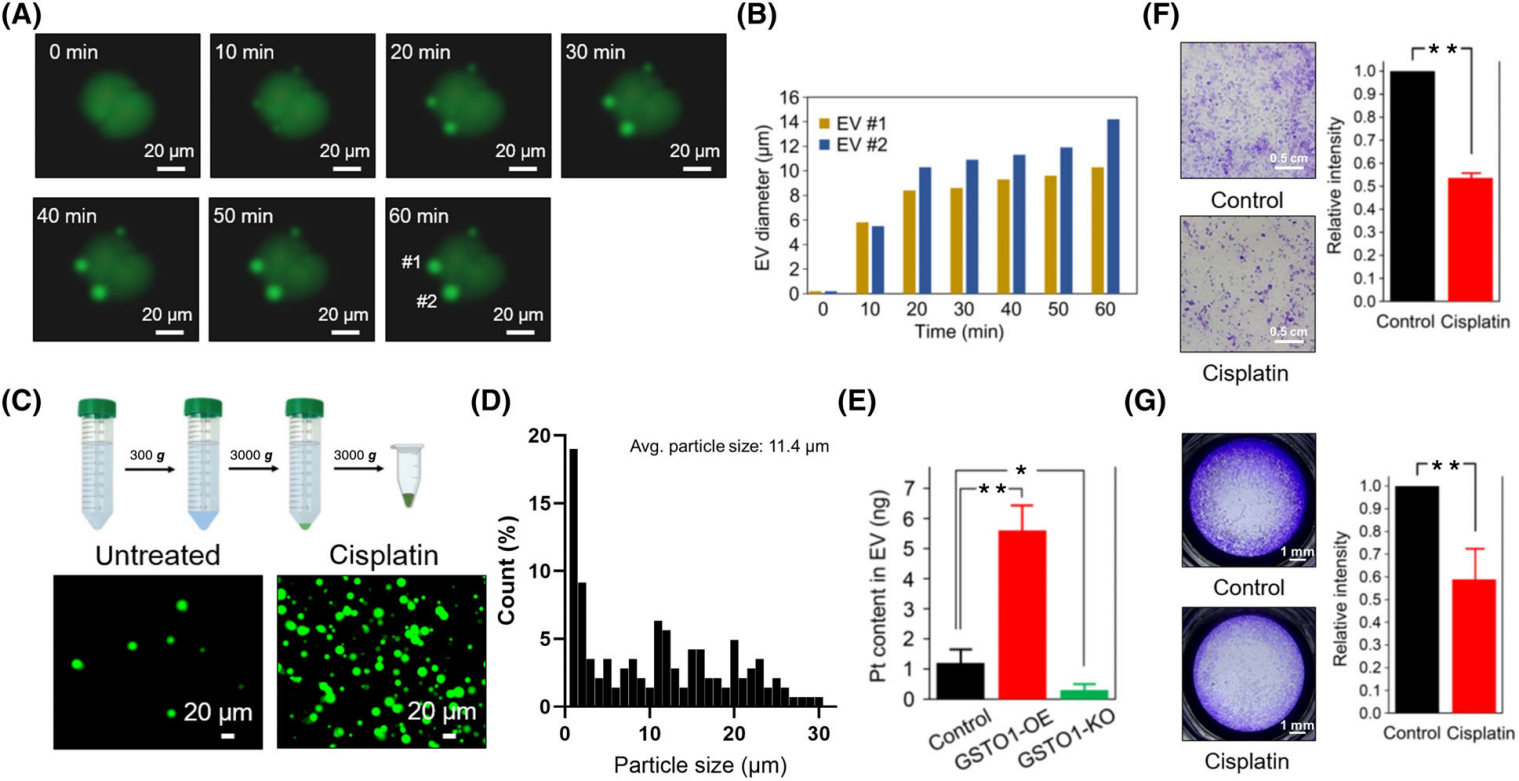
GSTO1‐containing EVs exhibit antitumor properties. (A) After cisplatin (5 μm) treatment, the formation of EVs in GSTO1‐OE HT1376 was monitored by time‐lapse fluorescence microscopy (*n* = 2). #1 and #2 are referred to as secreted EV1 and EV2. (B) The diameters of #1 and #2 EVs at various time intervals were measured and summarized (*n* = 2). (C) A differential centrifugation process for EV purification from conditional media: the centrifugation speeds of 300 ***g*** and 3000 ***g*** were used for cell debris removal and EV precipitation, respectively. After PBS washing once to remove contamination, EVs were collected and observed by fluorescence microscopy (*n* = 3). (D) The particle size was calculated and quantified by gene5 image ^+^ and the average particle size is 11.4 μm (*n* = 142). (E) After purification from conditional media (10 mL), the Pt content in EVs derived from different tumor cells was determined by ICP‐MS (*n* = 3). (F) Colony formation assay and (G) transwell migration assay were performed in EVs‐treated HT1376, where EVs derived from conditional media of tumor cells with or without cisplatin treatment were defined as the Cisplatin group and the Control, respectively. The signal quantification of crystal violet extract was measured by colorimetric analysis at 570 nm (right panel) (*n* = 3). Data were displayed as the mean ± SD. *P* values were calculated using a two‐tailed unpaired Student's *t*‐test (E–G). *P < 0.05; **P < 0.01. EVs, extracellular vesicles.

Time‐lapse observation of EV formation in bladder cancer cells expressing GSTO1‐GFP was performed to characterize GSTO1‐associated EVs. GSTO1‐containing EVs were formed within 2 h after cisplatin treatment, and the process of EV blebbing took approximately 1 h. The newly formed blebs were predominantly 10–20 μm in diameter (Fig. 5A,B). Due to their large size, the GSTO1‐containing EVs could be effectively purified using a low centrifugation speed of 3000 ***g*** (Fig. 5C). The quantity of EVs isolated from the media of cisplatin‐treated GSTO1‐OE cells was relatively higher than the quantity from the untreated control (Fig. 5C), and the average particle size was 11.4 μm (Fig. 5D). To further confirm that the GSTO1‐associated EVs were responsible for Pt efflux, we determined the Pt content in purified EVs. EVs derived from GSTO1‐OE cells had a significantly higher Pt content than EVs derived from control cells (Fig. 5E). In contrast, the Pt content was significantly lower in purified EVs derived from GSTO1‐KO cells (Fig. 5E), suggesting that GSTO1‐associated EVs contributed to Pt efflux from bladder cancer cells. Previous studies indicated that large EVs (oncosomes), with a size range of 1–10 μm, shed from tumor cells commonly exhibit pro‐tumorigenic activities [45]. To verify whether GSTO1‐associated EVs have properties similar to those of oncosomes, bladder cancer cells were treated with GSTO1‐associated EVs. Unexpectedly, GSTO1‐associated EVs attenuated the migration and colony formation of bladder cancer cells (Fig. 5F,G). These results imply that, unlike oncosomes, GSTO1‐associated EVs exhibit antitumor properties, likely through the export of hazardous materials.

**Fig. 6:**
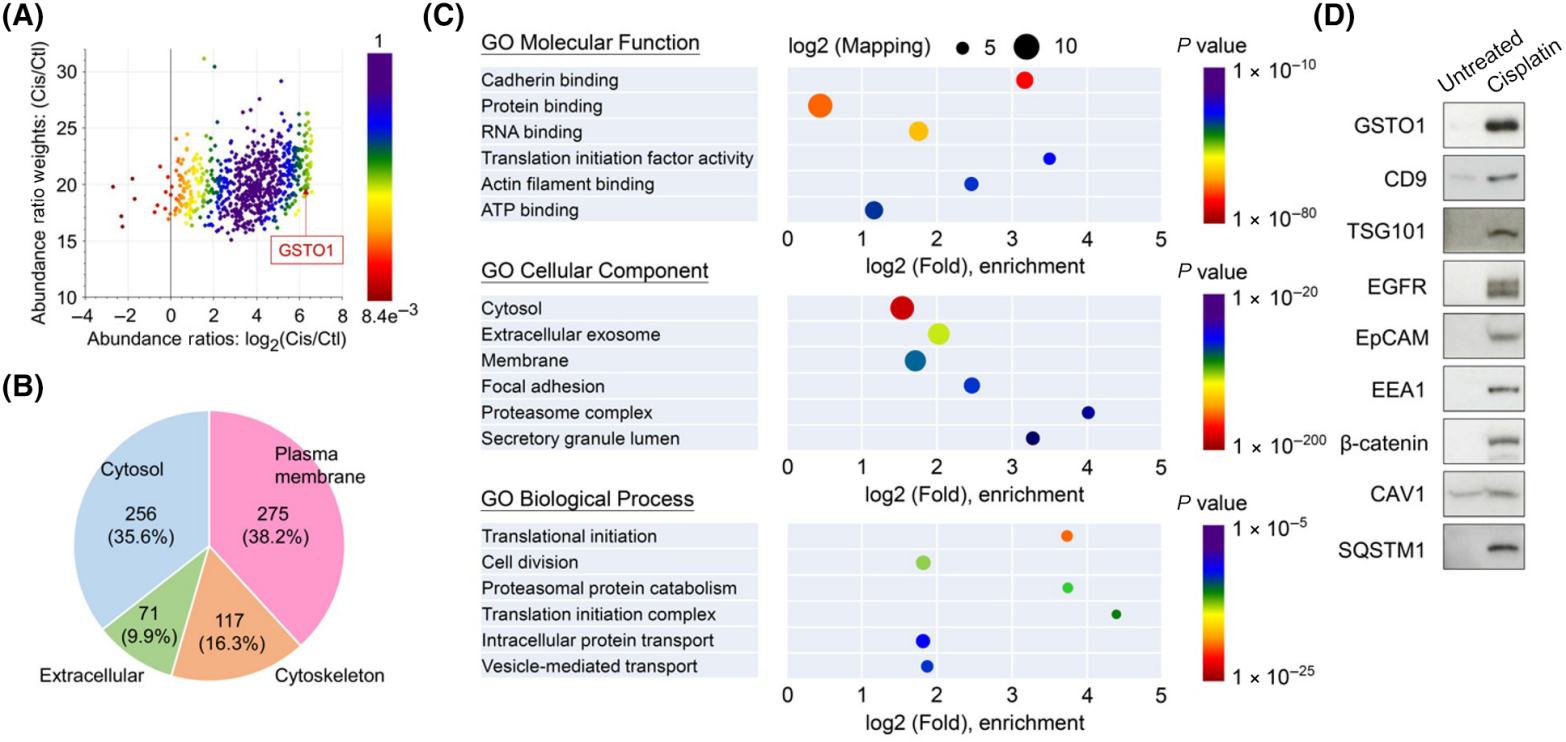
GSTO1‐containing EVs comprise common proteomic components in both small and large EVs. (A) The proteome of EVs derived from cisplatin‐treated and untreated (the control) HT1376 cells was analyzed by MS‐based identification. The scatter plot revealed the relationship between ratio weights (weighting by mass intensity) and abundance ratios of each protein between both samples (*n* = 1). The color of the protein dot represented the *P* value for abundance ratios adjusted by the background *t*‐test. (B) Cellular location analysis of the GSTO1‐containing EV proteome (*n* = 1). (C) Gene ontology (GO) pathway enrichment analyses for molecular function, cellular components, and biological processes were performed by the proteome discoverer software (Thermo Fisher Scientific). The size of each circle indicated the log base 2 of the counting number on each part, while the color represented the *P* value of the enrichment analysis (*n* = 1). (D) Western blot validation of the expression of the indicated protein in EVs of the cisplatin‐treated and control groups (*n* = 3). EVs, extracellular vesicles.

The proteomic composition of small EVs (30–150 nm), such as exosomes, has been well studied [46]. Large EVs (100–1000 nm) with potential pathological and therapeutic roles have recently attracted research attention [47]. To decipher the protein signature of GSTO1‐associated EVs, we determined the proteomic constitution of purified EVs using MS‐based technology. Except for ribosomal protein complexes and T‐complex proteins that are viewed as non‐vesicular protein contamination [48], a total of 719 proteins were identified in the GSTO1‐associated EV proteome (Fig. 6A; Table S3), which were mainly derived from the plasma membrane (*n* = 275, 38.2%), cytosol (*n* = 256, 35.6%), cytoskeleton (*n* = 117, 16.3%), and extracellular region (*n* = 71, 9.9%) (Fig. 6B; Table S3). The GO annotation analyses revealed that the enriched proteins were located in extracellular exosomes and secretory granule lumens and involved in intracellular protein transport and vesicle‐mediated transport, as well as mediation of cell–cell adhesion (Fig. 6C). Western blot validation showed that the GSTO1‐containing EVs highly expressed the exosome markers CD9 and TSG101 and the well‐known large EV proteins EGFR, EpCAM, and EEA1 [44] (Fig. 6D). In addition, the cadherin‐binding activities‐related proteins β‐catenin and CAV1 were significantly expressed in the GSTO1‐containing EVs [49] (Fig. 6D). Moreover, a previous study and the present study identified SQSTM1 (p62) in EVs, which was implicated in the linkage between the autophagy pathway and EVs for cargo release [50]. These results show that GSTO1‐associated EVs are larger in size (10–20 μm in diameter) than previously observed large EVs and exhibit a complex type of proteomic composition mixing the proteins identified from EVs classified as small or large.

### 3.6 GSTO1 contributes to the cisplatin resistance of bladder cancer *in vivo*

**Fig. 7:**
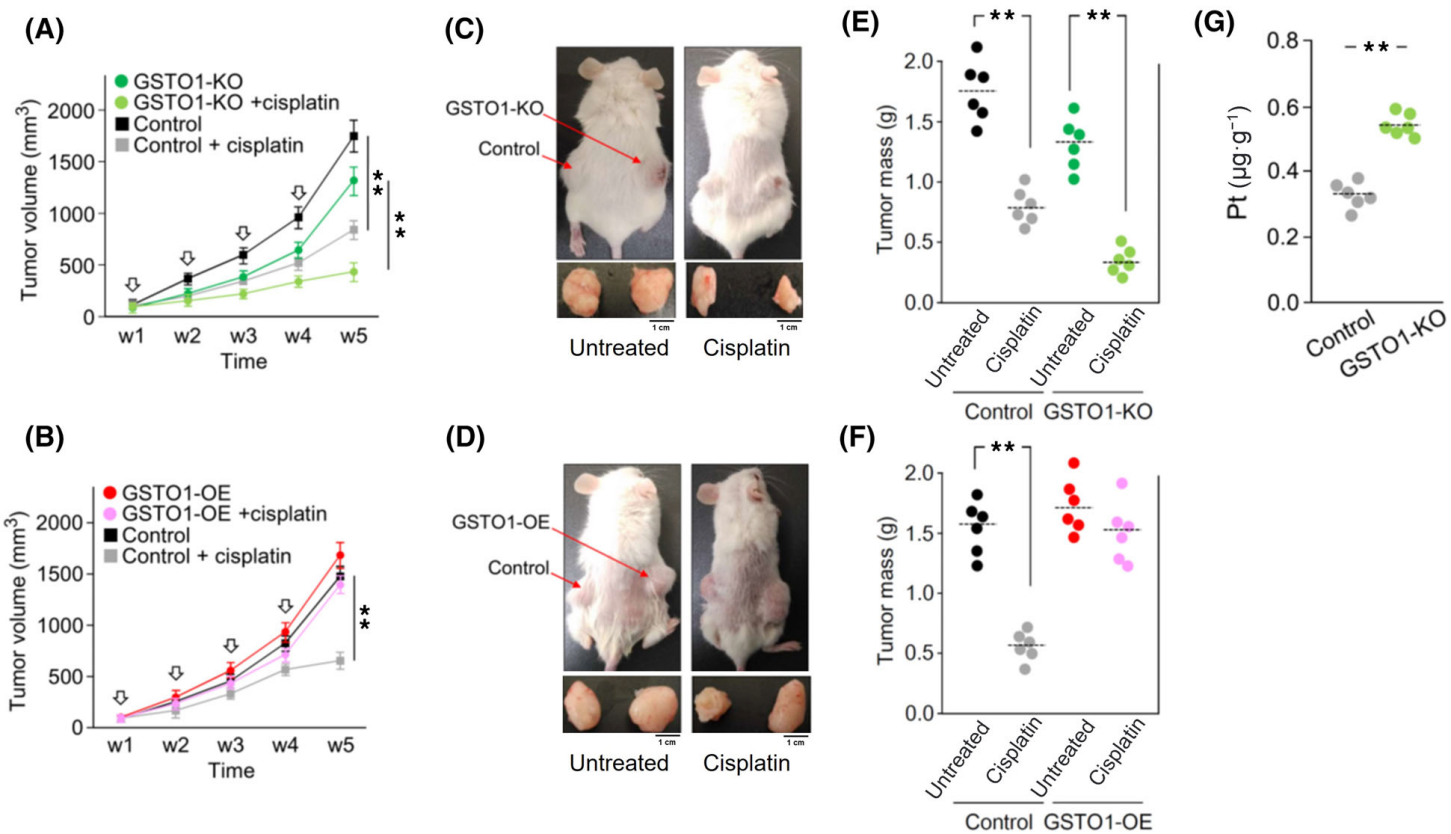
GSTO1 enhances the resistance of bladder cancer to cisplatin *in vivo*. Male SCID mice (5 weeks of age; *n* = 6) were subcutaneously injected with (A) GSTO1‐KO and the control HT1376 (1 × 10^6^ cells), or (B) GSTO1‐OE and the control HT1376 (1 × 10^6^ cells) into the right and left flanks, respectively. Until tumor volume was approximately 100 mm^3^ (defined as week 1, w1), mice were intravenously treated with cisplatin (3 mg·kg^−1^, 4 times/week) or PBS solution (untreated group). A total of four treatment cycles were conducted, which were indicated by arrow symbols. Tumor volumes were determined once per week. (C, D) showed the representative images of experimental mice and the corresponding tumors in both flanks at w5. (E, F) showed the weight of tumor masses in the indicated group of mice with or without cisplatin treatment. (G) The intracellular platinum (Pt) levels in the tumor masses of GSTO1‐KO and the control groups were determined by ICP‐MS. Data were displayed as the mean ± SD (A, B, E–G). *P* values were calculated using a two‐tailed, unpaired Student's *t*‐test. **P < 0.01. SCID, severe combined immunodeficiency.

To further evaluate the impact of GSTO1 on the efficacy of cisplatin treatment *in vivo*, mouse tumor models established with GSTO1‐OE and GSTO1‐KO cells were used. GSTO1‐OE/GSTO1‐KO and their control cells were subcutaneously injected into the right and left flanks of immune‐deficient mice, respectively. When tumor volume was approximately 100 mm^3^, mice were intravenously treated with cisplatin (3 mg·kg^−1^·week^−1^) and sacrificed at week 5. The statistical analyses of tumor volumes showed that GSTO1‐KO significantly sensitized tumor cells to cisplatin treatment compared to parental cells (Fig. 7A), whereas GSTO1‐OE dramatically abolished the efficacy of cisplatin in tumor control (Fig. 7B). After sacrifice, the tumor masses were weighted; the corresponding tumors in both flanks are presented in Fig. 7C,D. The tumor masses were significantly reduced in the GSTO1‐KO group subjected to cisplatin treatment, whereas cisplatin had no obvious effect on the GSTO1‐OE group compared to the control (Fig. 7E,F). In addition, the ICP‐MS analysis showed that the Pt content was significantly higher in the GSTO1‐KO tumor tissue than in the tumor tissue of the control (Fig. 7G). Collectively, the *in vivo* animal assay supported our findings that GSTO1 enhanced cisplatin resistance through the promotion of cisplatin efflux from bladder tumor tissue.

## 4 Discussion

Muscle‐invasive bladder cancer represents approximately 20% of newly diagnosed cases of bladder cancer, and the 5‐year OS rate of patients with MIBC is approximately 60–70% [51]. Neoadjuvant cisplatin‐based chemotherapy was designed as a potential strategy for MIBC treatment in the 1980s [52]. The BA06 30894 trial, the largest study of neoadjuvant chemotherapy completed to date, revealed 30–36% improvement in 10‐year survival (HR = 0.84; 95% CI = 0.72–0.99; *P* = 0.037) [53]. An updated meta‐analysis of 11 clinical trials consisting of > 3000 patients indicated a 5% 5‐year OS benefit and a 14% decrease in mortality risk (HR = 0.86; 95% CI = 0.77–0.95; *P* = 0.003) in advanced bladder cancer patients treated with neoadjuvant chemotherapy [54]. Thus, neoadjuvant cisplatin‐based chemotherapy has become the standard of care for eligible patients with MIBC. However, the benefit of neoadjuvant cisplatin‐based chemotherapy is limited to only a subset of patients [55], and acquired resistance still remains the major challenge to treatment efficacy. Several mechanisms have been implicated in cisplatin resistance, including decreased cisplatin uptake, increased cisplatin efflux, increased conjugation between cisplatin and glutathione, and activation of the DNA repair system [5]. Through large‐scale next‐generation analyses, DNA repair genes such as *ERCC2*, *ATM*, *RB1*, and *FANCC* were identified as biomarkers for predicting therapeutic response [56, 57]. However, the clinical validity and precise utility of these biomarkers for special subtypes of MIBC patients should be further evaluated. In this study, we found that TAM infiltration could enhance the resistance of bladder cancer to cisplatin through TNF‐α secretion. TNF‐α is a multifunctional cytokine mainly secreted by TAMs in the TME [58]. TNF‐α has been shown to promote chemoresistance through several mechanisms, including activation of the NF‐κB pathway, regulation of drug efflux, induction of the cancer stem cell phenotype, and modulation of the TME [59]. This study found that TNF‐α was responsible for activating GSTO1 expression in macrophage–tumor coculture condition media. GSTO1‐OE increased cisplatin resistance, whereas GSTO1‐KO or the GSTO1 inhibitor GSTO1‐IN‐1 sensitized bladder cancer to cisplatin (Fig. 3D–F). In addition, the expression of GSTO1 was increased in cisplatin‐resistant bladder cancer cells (Fig. 3C). These results suggest that GSTO1 may serve as a potential biomarker for predicting the response of bladder cancer to cisplatin.

The GSTs, a diverse family of cytosolic, mitochondrial, and microsomal enzymes, are classified into seven classes: alpha, mu, pi, theta, sigma, zeta, and omega. In humans, omega GST (GSTO), which contains two isozymes, GSTO1 and GSTO2, belongs to an atypical cytosolic class. Instead of the tyrosine or serine residue, GSTO1 has a cysteine residue in its active site that leads to a loss of GST activity while conferring a series of unusual activities, including the reaction of thioltransferase and the reduction of dehydroascorbate and monomethylarsonate (V) [8]. In addition, recent studies implicate critical roles for GSTO1 in pro‐inflammatory and oxidative stress in diverse types of cells, including immune cells and tumor cells [39]. GSTO1 overexpression has been found in several cancers, such as esophageal cancer, lung cancer, breast cancer, kidney cancer, and bladder cancer [11, 12, 13, 14]. The function of GSTO1 in chemoresistance was originally recognized via proteomic analysis by comparison between cisplatin‐resistant and parental tumor cells [15]. The mechanism of GSTO1's involvement in cisplatin resistance is suggested to be through activation of the Akt and ERK1/2 pathways to promote cell survival and JNK inhibition to block apoptosis [17, 60]. In the present study, we found that GSTO1 reduced intracellular cisplatin accumulation, thereby resulting in the attenuation of DNA–cisplatin adduct formation (Fig. 4A). Instead of ABC transporters, GSTO1 increases the efflux of chemotherapeutic drugs through the activation of EV release. This is the first identification of a novel function of GSTO1, which strongly implicates GSTO1 as a potential target for chemotherapeutic sensitization [18, 61].

Currently, EVs are classified into two categories: exosomes and microvesicles [62]. When multivesicular endosomes fuse with the plasma membrane, intraluminal vesicles, or exosomes, are released within the lumen of multivesicular endosomes. Exosomes, typically 30–100 nm in diameter, are the most studied type of EV. Microvesicles are EVs formed by the budding of the plasma membrane and range in diameter from 50 to 1000 nm, but can even be up to 10 μm. EVs were initially described as having the function of eliminating unneeded compounds from cells [63]; their capacities to exchange components between cells and to act as signaling vehicles are now recognized [62]. Tumor cells have been identified to shed a heterogeneous mixture of EVs, which are implicated in diverse physiological processes. Oncosomes (1–10 μm) are characterized as membrane‐derived large microvesicles that are released from tumor cells to transfer oncogenic signals to recipient cells, promote metabolic rewiring and the metastatic cascade, and reshape the TME [64].

Accumulating data reveal that oncosomes could function as part of a mechanism of acquired chemoresistance via the transfer of multidrug resistance protein‐1 and multidrug resistance‐associated protein‐1 from drug‐resistant to recipient tumor cells. In this study, we found that although GSTO1‐associated EVs were similar in size to oncosomes, their properties were quite different. Cisplatin‐induced GSTO1‐associated EVs showed anti‐cancer activities by dramatically impairing tumor colony formation and migration abilities (Fig. 5D,E). Several multidrug transporters have been identified on the membrane of large oncosomes, which contribute to the efflux of cisplatin from the oncosomes [23]. These transporters were not detected on the GSTO1‐associated EVs (Table S2). This absence of transporters could potentially result in the containment of cisplatin within EVs, thereby conferring anti‐cancer properties to the EVs. However, the functions of GSTO1‐associated EVs in the recipient cells remain to be characterized in detail.

## 5 Conclusion

**Fig. 8:**
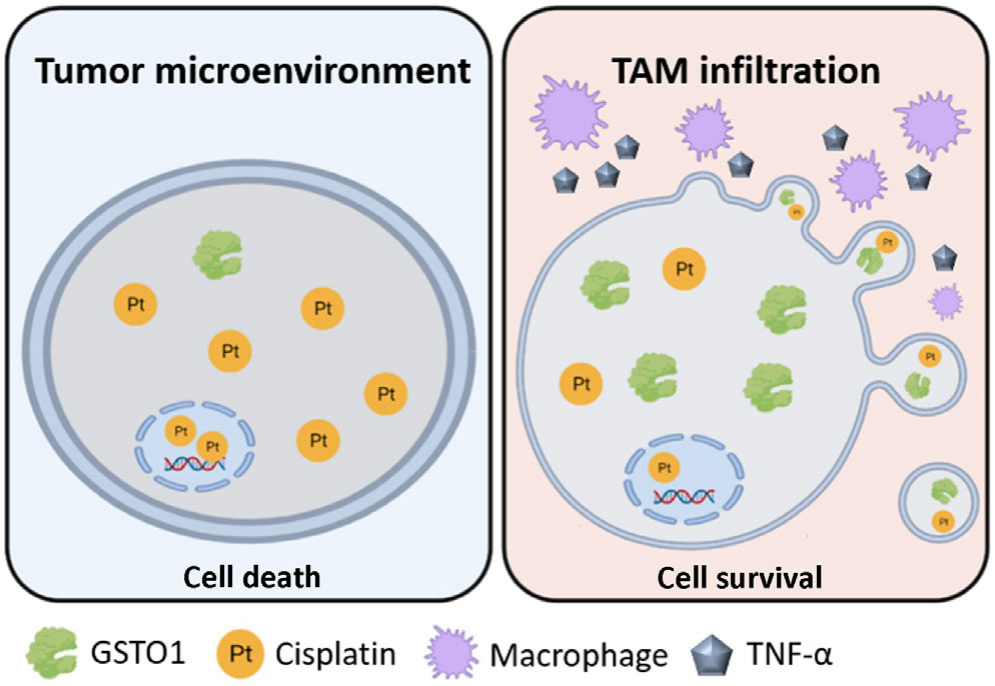
The representative working model in this study. In the tumor microenvironment, TAM infiltration induces the overexpression of GSTO1 in bladder cancer. GSTO1 activates EV release for cisplatin efflux, finally leading to chemotherapeutic resistance. EV, extracellular vesicle; TAM, tumor‐associated macrophage.

The present study showed that secretory TNF‐α from TAMs induced GSTO1 expression in bladder cancer cells, which in turn contributed to the generation of large EVs for cisplatin efflux. This effect ultimately led to increased cisplatin resistance in bladder cancer, as shown in Fig. 8. The development of strategies that target GSTO1 may hold promise for improving therapeutic efficacy against bladder cancer in the future.

## Conflict of interest

The authors declare no conflict of interest.

## Author contributions

Y‐CP, W‐CC, and Y‐CW conceived and designed the key experiments; Y‐CP performed most of the experiments and wrote this manuscript; P‐YC and W‐YH performed animal experiments. C‐CL and C‐YH provided the bladder cancer tissue specimens. L‐FS, J‐CY, W‐CC, and Y‐CW provided suggestions for the experimental design and revised this manuscript. All authors have read and agreed to the published version of the manuscript.

## Supporting information


**Table S1.** Proteomic alterations within bladder cancer under the CM treatment.

**Table S2.** The proteins of CM‐induced chemoresistance in bladder cancer.

**Table S3.** GSTO1‐associated EV proteome.

## Data Availability

Data will be made available on request.
